# Spontaneous Polarization Suppression of Exciton–Exciton Annihilation in Rhombohedral-Stacked Bilayer Molybdenum Disulfide

**DOI:** 10.1021/acsnano.6c05069

**Published:** 2026-06-25

**Authors:** Tae Gwan Park, Xufan Li, Kyungnam Kang, David B. Geohegan, Christopher M. Rouleau, Alexander A. Puretzky, Kai Xiao

**Affiliations:** † Center for Nanophase Materials Sciences, 518778 Oak Ridge National Laboratory, Oak Ridge, Tennessee 37831, United States; ‡ Center for Integrated Nanotechnologies, Los Alamos National Laboratory, Los Alamos, New Mexico 87544, United States; § 140422 Honda Research Institute USA Inc., San Jose, California 95134, United States; ∥ Department of Materials Science and Engineering, 122608 University of Tennessee at Knoxville, Knoxville, Tennessee 37996, United States

**Keywords:** exciton−exciton annihilation, rhombohedral stacking, molybdenum disulfide, spontaneous polarization, dipolar excitons, two-dimensional semiconductors, ultrafast spectroscopy

## Abstract

Rapid exciton–exciton annihilation (EEA) in two-dimensional semiconductors limits access to high-density excitonic regimes essential for efficient optoelectronic operation under strong excitation. Here, we show that EEA is strongly suppressed in rhombohedral (3R)-stacked MoS_2_ bilayers relative to nonpolar 2H bilayers, and that this suppression is quantitatively consistent with repulsive dipole–dipole interactions between layer-polarized excitons induced by spontaneous polarization. Using ultrafast pump–probe spectroscopy, we measure an EEA rate of γ_EEA_ = (5.03 ± 0.99) × 10^–3^ cm^2^ s^–1^ in 3R bilayers, which is approximately 18.2-fold smaller than that in monolayers and 2.9-fold smaller than that in nonpolar 2H bilayers. Despite the higher exciton diffusivity recently reported for 3R relative to 2H bilayers, the reduced EEA rate in 3R indicates a rate-limited regime governed by the close-encounter annihilation probability rather than diffusion. A rate-limited annihilation model incorporating a dipole–dipole repulsive potential captures the observed ratio γ_EEA,3R_/γ_EEA,2H_ ≈ 0.35 for an exciton–exciton encounter distance of ∼1.3 nm, consistent with the bilayer exciton Bohr radius. These results indicate that spontaneous polarization in 3R-stacked bilayers can suppress nonlinear excitonic losses and provide a route toward high-density excitonics.

## Graphical abstract

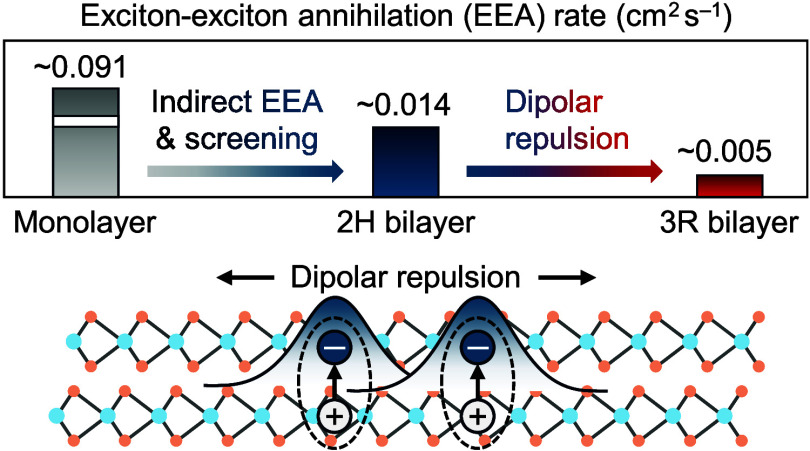

## Introduction

Reduced dielectric screening and quantum confinement in atomically thin semiconductors markedly enhance Coulomb interactions, stabilizing tightly bound excitons with large binding energies and strong light-matter coupling.^1,2^ These attributes make two-dimensional (2D) transition metal dichalcogenides (TMDs) a compelling materials platform for photonics and optoelectronics^1,3–5^ —such as photodetectors, light emitters, and nonlinear optical devices—enabled by large oscillator strengths and robust excitonic resonances that persist even at room temperature. However, the same low-dimensionality also strengthens many-body interactions among photoexcited quasiparticles. A prominent example is exciton–exciton annihilation (EEA), in which two excitons interact such that one recombines nonradiatively while transferring its energy and momentum to the other, a process that becomes significant at relatively high exciton densities. As excitation densities increase, exciton–exciton encounters become more frequent, activating efficient nonradiative loss pathways that define the upper limit of sustainable exciton density and the efficiency of photonic and optoelectronic devices.^6–8^ In 2D TMDs, EEA is typically much more efficient than in bulk counterparts and conventional 3D semiconductors, introducing a pronounced density-dependent decay channel that rapidly depletes the exciton population and highlights the challenge of sustaining high-density, long-lived excitonic states.^6,8–14^

Mitigating EEA is therefore crucial not only for efficient optoelectronic operation across a wide range of excitation densities^7,15,16^ but also for accessing strongly correlated excitonic phases in 2D semiconductors.^17–21^ Reported strategies include detuning band-structure resonances through strain,^7,15,16^ modifying Coulomb screening via dielectric engineering,^22,23^ and exploiting metasurfaces^20,24^ and van der Waals heterostructures.^25^ In particular, spatial separation of carriers through formation of dipolar excitons, e.g., interlayer excitons with out-of-plane dipole moments in bilayer semiconductors, can alter excitonic transport and many-body dynamics through dipole–dipole repulsion.^26–30^ Such repulsive interactions could, in principle, introduce an effective interaction barrier that suppresses the short-range exciton–exciton encounters required for annihilation, but their quantitative impact on EEA remains largely unexplored.

Rhombohedral (3R) MoS_2_ bilayers intrinsically host dipolar excitons via spontaneous out-of-plane polarization.^31–33^ In 3R stacking, broken inversion symmetry produces an intrinsic interlayer potential that layer-polarizes K valley electronic states, reduces electron–hole overlap, and yields an out-of-plane exciton dipole moment,^30–34^ offering a route to investigate the role of dipolar repulsion in EEA. Despite extensive studies of exciton dynamics in 3R MoS_2_ bilayers,^13,30,34–36^ isolating and quantifying the contribution of dipolar repulsion to EEA remains experimentally challenging. First, experimentally resolving EEA requires an intermediate excitation density, above the low densities where bimolecular annihilation is negligible^34,36^ but below sufficiently high densities at which competing ultrafast channels, such as defect-assisted or Auger recombination processes, obscure EEA in time-domain measurements.^35^ Second, quantitative attribution to dipolar repulsion cannot rely on monolayer–bilayer comparisons, because bilayer TMDs introduce additional layer-number effects, most notably indirect-gap relaxation channels^35^ and associated nonradiative pathways,^10,13^ that can modify exciton lifetimes. A more appropriate control is the centrosymmetric 2H bilayer, which shares the same chemical composition and thickness but lacks built-in polarization. Third, EEA is highly sensitive to extrinsic factors such as dielectric screening,^11,12^ local strain,^7,15,16^ and defect density,^37–39^ necessitating a side-by-side comparison of 2H and 3R bilayers with closely matched crystalline quality and dielectric environment. Finally, while encapsulation with h-BN is often employed to improve optical properties, it can itself suppress EEA through enhanced dielectric screening and reduced disorder,^22,23^ thereby making EEA difficult to identify even at relatively high excitation densities.^30^ These constraints have so far hindered an investigation of how spontaneous polarization and the resulting dipolar repulsion modulate the EEA rate in 3R-stacked bilayers.

In this work, we experimentally resolve a clear stacking-dependent suppression of EEA by comparing 2H and 3R MoS_2_ bilayers grown on the same substrate and confirmed to have comparable quality. By carefully probing fluence-dependent transient reflectance within an intermediate pump-fluence range, we directly compare EEA rates in 2H and 3R MoS_2_ bilayers and find that the EEA rate in 3R bilayers is ∼2.9-fold smaller than that in 2H. Although 3R bilayers exhibit higher exciton diffusivity than 2H bilayers,^30^ the reduced EEA in 3R bilayers indicates a rate-limited regime where the close-encounter annihilation probability, rather than diffusion, controls the EEA rate. A rate-limited annihilation model incorporating short-range repulsive dipole–dipole interactions between layer-polarized excitons provides a quantitatively consistent interpretation of the observed suppression, supporting a role for spontaneous polarization in governing exciton–exciton interactions. These results show that 3R stacking and the associated built-in polarization provide an intrinsic route to suppress nonlinear excitonic losses and expand the accessible high-density and long-lived exciton regime in 2D semiconductors.

## Results and Discussion

### Structural and Electronic Properties of 2H and 3R Bilayer MoS_2_

Both hexagonal 2H and rhombohedral 3R bilayers occur in natural deposits.^40,41^ As illustrated in Figure 1(a), the 2H bilayer comprises two layers rotated by 180° relative to each other, which restores inversion symmetry for an even number of layers. In contrast, in 3R bilayers [Figure 1(b)], adjacent layers share the same crystallographic orientation but are laterally shifted by one-third of the unit cell (i.e., the metal sublattice in one layer aligns above the chalcogen sublattice in another layer). This breaks inversion symmetry and gives rise to an out-of-plane spontaneous polarization.^31–33^ The lack of inversion symmetry in 3R bilayers enables nonlinear optical response,^42,43^ ferroelectric behavior,^33,44,45^ ultrafast spontaneous photovoltaic effect,^46^ and nanosecond ferroelectric switching of intralayer excitons.^47^ These symmetry differences also produce distinct electronic band structures as shown in Figure 1(c). In 3R bilayers, the built-in polarization generates an intrinsic interlayer potential and asymmetric interlayer coupling that lifts the layer degeneracy at the K valleys, effectively resulting in a type-II alignment.^31,32^ By contrast, in 2H bilayers, the K valley conduction and valence bands remain layer-degenerate and show no net layer selectivity in the electronic eigenstates.^30,34^

**Figure 1:**
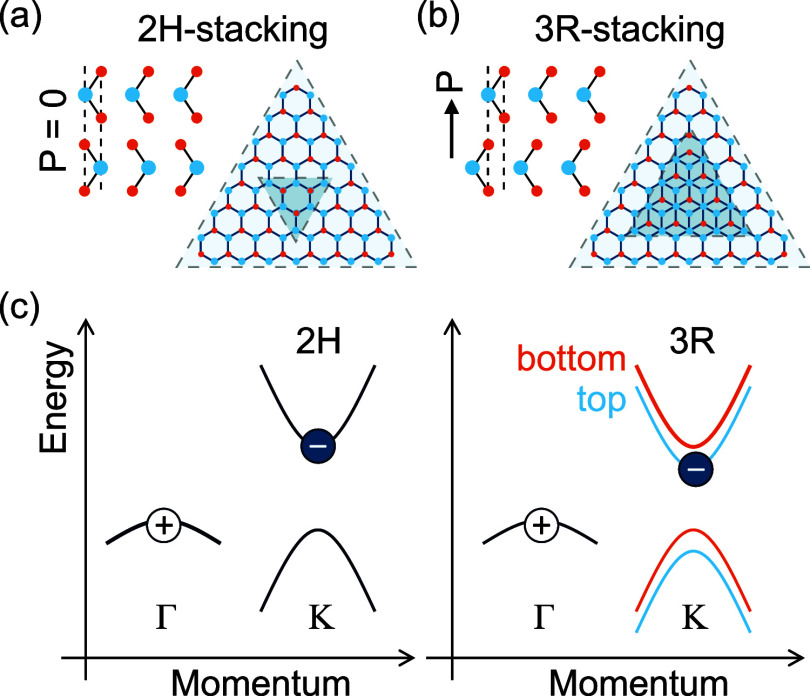
Stacking-dependent symmetry and electronic structure of MoS_2_ bilayers. (a) 2H (AA′) stacking with 180° rotation between layers, restoring inversion symmetry and yielding zero out-of-plane polarization (*P* = 0). (b) 3R (AB) stacking with a lateral shift of one-third of the unit cell, breaking inversion symmetry and inducing an out-of-plane polarization (*P* ≠ 0; opposite for BA stacking). (c) Schematic band structure of 2H and 3R bilayers.

Because bilayer TMDs are indirect-gap semiconductors, the valence-band maximum shifts toward the Γ valley. In 3R MoS_2_ bilayers, photoexcited electrons in the K valley become layer-selective and preferentially occupy the lower-energy K-point conduction-band edge of one layer (top layer, for AB stacking), while holes accumulate near the Γ-valley valence-band edge [Figure 1(c)].^34^ Meanwhile, the valence states at the Γ point remain strongly hybridized across layers, which reduces hole layer polarization relative to electrons. The resulting layer-selective electronic structure enhances electron localization to a specific layer in 3R, reduces electron–hole wave function overlap, and imparts dipolar exciton character. Consistent with this picture, prior ultrafast spectroscopy studies have reported prolonged exciton lifetimes^34^ and superdiffusive exciton transport^30^ in 3R-stacked MoS_2_ bilayers.

### Sample Preparation and Optical Characterization

Bilayer MoS_2_ crystals were grown on SiO_2_/Si substrates by chemical vapor deposition (CVD) as described in the Methods section. Because the thermodynamically stable bilayer stacking configurations of MoS_2_ are 2H and 3R,^40,41^ the as-grown samples naturally contain both 2H- and 3R-stacked bilayer domains on a single substrate. The stacking configuration was identified from optical microscope images of as-grown bilayer domains, based on the relative rotation angles formed during CVD, as shown in the insets of Figure 2(a). The typical edge length of the top layer in the bilayer region is ∼20 μm. Importantly, the coexistence of large-area, uniform 2H and 3R bilayers on one substrate enables direct side-by-side optical comparisons under identical experimental conditions.

**Figure 2:**
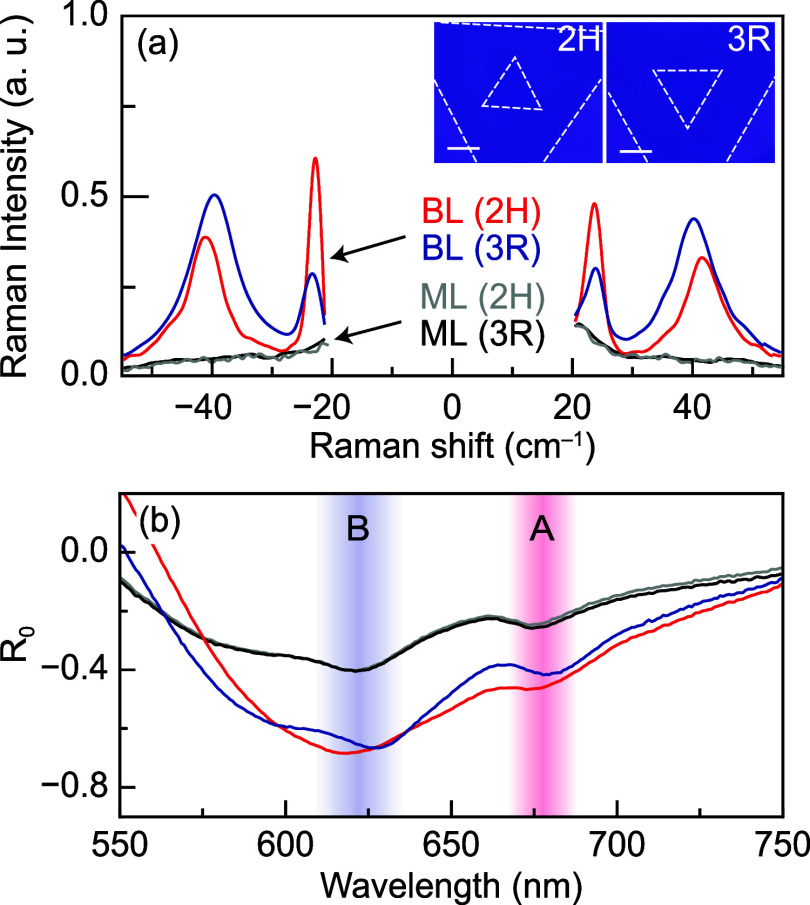
Optical identification of stacking configuration in MoS_2_ bilayers. (a) Low-frequency Raman spectra of monolayer (ML) and bilayer (BL) 2H and 3R MoS_2_, labeled ML (2H), ML (3R), BL (2H), and BL (3R). Two interlayer vibrational modes are observed in the BL samples: the in-plane shear mode at ∼24 cm^–1^ and the out-of-plane breathing mode at ∼40–42 cm^–1^. Insets: Optical microscope images of 2H and 3R samples. The inner white dashed triangle outlines the BL region, while the area up to the outer white dashed boundary corresponds to the ML region. Scale bars: 10 μm. (b) Corresponding normalized reflectance spectra. Shaded regions indicate the A and B excitonic resonances.

Optical characterization was performed using Raman spectroscopy and optical reflectance at room temperature under ambient conditions. Raman spectra were acquired using a 532 nm continuous-wave laser (beam spot ∼ 1 μm), and reflectance spectra were measured using a white-light continuum source (beam spot ∼ 5 μm) (see Methods section). Low-frequency Raman spectroscopy further confirms the stacking configuration [Figure 2(a)]. In the low-frequency range (<60 cm^–1^), two pronounced peaks appear only in bilayer MoS_2_ and are assigned to the interlayer shear (SM) and breathing (BM) modes, consistent with previous observations.^48,49^ The SM lies at ∼24 cm^–1^ for both 2H and 3R bilayers, while the BM peak occurs at higher frequency in 2H bilayers (∼42 cm^–1^) than in 3R bilayers (∼40 cm^–1^). The softening of the BM in 3R indicates a slightly weaker interlayer restoring force compared to 2H, consistent with stronger interlayer coupling in 2H.^48,49^ Full Raman spectra are provided in Figure S1 in the Supporting Information.

Figure 2(b) shows the normalized reflectance spectrum, *R*_0_ = *R*_sample_/*R*_substrate_, from monolayers (ML) and 2H- and 3R-stacked bilayers, where the excitonic resonances appear as pronounced spectral features. For MoS_2_ on SiO_2_/Si substrate, *R*_0_ typically shows dips near the exciton resonances due to absorption- and phase-induced changes in MoS_2_ combined with interference effects in the SiO_2_ layer.^50^ Across the monolayer, 2H, and 3R bilayer samples, two resonances near ∼680 and 625 nm correspond to the A and B excitons, respectively. Spectra acquired from monolayer regions adjacent to the 2H and 3R bilayer domains are nearly identical, indicating similar crystal quality. Compared with ML, bilayers display stronger and spectrally modified excitonic responses. Subtle differences between 2H and 3R bilayers are also observed, reflecting stacking-dependent interlayer coupling and electronic structure.^41^

### Excitation Density-Dependent Ultrafast Pump–Probe Spectroscopy

Figure 3 summarizes the stacking-configuration- and excitation-density-dependent exciton kinetics in monolayer, 2H-, and 3R-stacked MoS_2_ bilayers measured by ultrafast pump–probe spectroscopy at room temperature under ambient conditions. Using a 400 nm pump and a broadband white-light continuum probe, we measured the transient reflectance, Δ*R*/*R*_0_, as a function of probe wavelength and pump–probe time delay (*t*), as shown in Figure 3(a–c). Both pump and probe spot sizes were ∼5 μm at the sample, and the pump fluence (*F*_pump_) was varied from ∼20 to 61 μJ cm^–2^ using a set of neutral-density optical filters (see Methods section for details). Over this *F*_pump_ range, the initial exciton density per layer is estimated as *n*_0_ = (1.0 – 3.1) × 10^12^ cm^–2^ by accounting for optical absorption of MoS_2_ and substrate interference effects (see Methods section) and assuming one absorbed photon creates one electron–hole pair.

**Figure 3:**
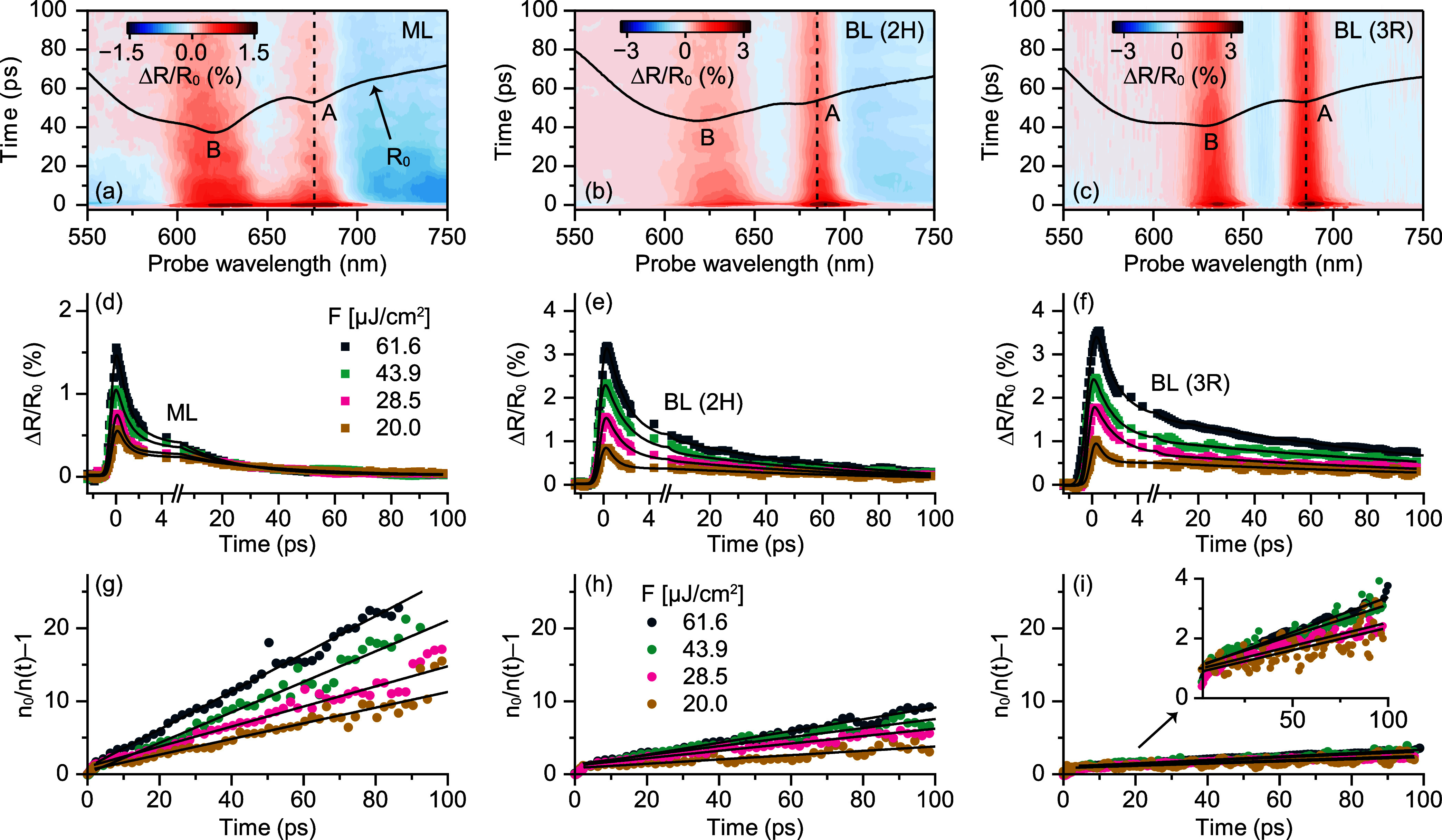
Ultrafast exciton dynamics of monolayer and bilayer MoS_2_ at varying pump fluence. (a–c) Transient reflectance maps, Δ*R*/*R*_0_, as a function of probe wavelength and pump–probe time delay for (a) monolayer, (b) 2H bilayer, and (c) 3R bilayer measured at a pump fluence of 61.6 μJ cm^–2^. The black curve shows the linear reflectance spectrum *R*_0_ in Figure 2(b) with arbitrary scaling. The vertical dashed lines indicate the A-exciton resonance. (d–f) Corresponding Δ*R*/*R*_0_ kinetics extracted at the A-exciton bleach wavelength at different pump fluences, with biexponential fits (black curves). (g–i) Corresponding time evolution of *n*_0_/*n*(*t*) – 1. Solid black lines are linear fits in the EEA-dominated regime (*t* ≳ 3 ps), with slopes *n*_0_γ_EEA_. The inset in (i) enlarges the 3R data for clarity.

For the monolayer in Figure 3(a), the signal was acquired from a monolayer region adjacent to the 2H bilayer domain. We confirmed that its pump–probe response is nearly identical to that of a monolayer region adjacent to the 3R domain (Figure S2). The black curves overlaid on the transient maps in Figure 3(a–c) are the linear reflectance spectra, *R*_0_ in Figure 2(b), plotted with arbitrary scaling as a visual guide to the excitonic resonance positions. As indicated by the vertical dashed lines, the positive Δ*R*/*R*_0_ signal near 680 nm is assigned to photoinduced bleaching of the K valley A-exciton resonance, and therefore it tracks the kinetics of the K valley carrier population, whose amplitude is approximately proportional to the exciton density.^30,34^ Figure 3(d–f) shows the excitation-density-dependent kinetics extracted at this probe wavelength. Across all samples, the peak Δ*R*/*R*_0_ scales approximately linearly with *F*_pump_ (Figure S3).

For each sample, the transients are well described by a biexponential decay. The fast and slow time constants (τ_1_ and τ_2_) extracted at each *F*_pump_ are summarized in Table S1 in the Supporting Information. In all samples, τ_1_ ≈ 1.0–1.5 ps, which we attribute primarily to rapid hot-exciton cooling and fast exciton capture by defects.^51–53^ The slower component, τ_2_, which dominates for *t* ≳ 3 ps after the τ_1_ process, is strongly modulated by both stacking and excitation density. In the monolayer, τ_2_ decreases from ∼37 ps at low excitation density (*F*_pump_ = 20 μJ cm^–2^) to ∼19 ps at high excitation density (*F*_pump_ = 61.6 μJ cm^–2^). In the 2H bilayer, τ_2_ decreases from ∼95 ps to ∼64 ps over the same fluence range. In the 3R bilayer, τ_2_ shows a more modest reduction, decreasing from ∼160 ps to ∼135 ps as *F*_pump_ increases from 20 to 61.6 μJ cm^–2^. Such reductions in τ_2_ with increasing excitation density are consistent with density-dependent nonradiative Auger processes, i.e., EEA.^6,10,11,13^ Notably, τ_2_ also depends strongly on stacking: at a fixed *F*_pump_, τ_2_ in 3R is nearly twice that in 2H. This longer lifetime in 3R is consistent with prior reports,^34^ which attributed the longer recombination lifetime to reduced electron–hole wave function overlap arising from the built-in polarization in 3R bilayers. The *F*_pump_-dependent kinetics across monolayer, 2H, and 3R enable a quantitative extraction of the EEA rate while disentangling layer-number and stacking effects, as discussed next.

### Exciton–Exciton Annihilation Rate

In the presence of EEA, the exciton population kinetics *n*(*t*) can be described by a rate equation for bimolecular recombination

1 $$\frac{\mathrm{dn}}{\mathrm{dt}}=-\gamma_{1}n-\gamma_{\mathrm{EEA}}n^{2}$$

where *n* is the exciton density (cm^–2^), γ_1_ is the first-order recombination rate constant (s^–1^), and γ_EEA_ is the EEA rate (cm^2^ s^–1^). At early times and sufficiently high densities such that γ_EEA_ *n* ≫ γ_1_ (typically within a few to 100 ps),^6,9,11,13,38,39^ the EEA term dominates, and the solution reduces to

2 $$\frac{n_{0}}{n\left(t\right)}-1=\gamma_{\mathrm{EEA}}n_{0}t$$

a form widely used to analyze EEA-driven decay dynamics.^6,9–11^ Figures 3(g–i) show the exciton kinetics in terms of *n*_0_/*n*(*t*) – 1. The data exhibit linear behavior for *t* ≳ 3 ps, i.e., after the fast τ_1_ process has substantially decayed, with slopes of γ_EEA_ *n*_0_, consistent with an EEA-dominated regime in this window. The extracted γ_EEA_ values were insensitive, within fitting uncertainty, to reasonable variations of the fit start time in the 3–5 ps range. Using eq 2 and *n*_0_, EEA rates are estimated as γ_EEA_ = (9.14 ± 0.85) × 10^–2^ cm^2^ s^–1^ for monolayer (ML), (1.43 ± 0.37) × 10^–2^ cm^2^ s^–1^ for the 2H bilayer, and (5.03 ± 0.99) × 10^–3^ cm^2^ s^–1^ for the 3R bilayer (Table S2). While the absolute γ_EEA_ values depend on the estimated *n*_0_, the observed suppression of γ_EEA_ in 3R relative to 2H is more robust because both bilayer domains were measured on the same substrate under identical optical conditions. The monolayer value is comparable to previously reported ranges of 4 × 10^–2^ to 7 × 10^–2^ cm^2^ s^–1^ for unencapsulated MoS_2_ monolayers.^6,11,14,54^ Differences among reported γ_EEA,ML_ values (including ours) are commonly attributed to dielectric screening,^11,12^ local strain,^7,15,16^ and defect density.^37–39^ These extrinsic factors are unlikely to dominate the present bilayer comparison because the 2H and 3R domains were grown on the same substrate and exhibit nearly identical optical characteristics; accordingly, adjacent monolayer regions show nearly identical EEA behavior (Figure S2), and we therefore do not invoke extrinsic differences to explain the bilayer trend.

Both bilayers exhibit markedly smaller slopes than the monolayer [Figure 3(g–i)], yielding reduced EEA rates: γ_EEA, 2H_ = (1.43 ± 0.37) × 10^–2^ cm^2^ s^–1^ and γ_EEA, 3R_ = (5.03 ± 0.99) × 10^–3^ cm^2^ s^–1^. Compared to the monolayer, γ_EEA_ is reduced by a factor of ∼ 6.4 in 2H and ∼18.2 in 3R. Suppressed EEA in bilayer TMDs compared with monolayers is well established,^10,13,14^ and is generally attributed to two effects: (1) enhanced dielectric screening in thicker layers, which weakens Coulomb-mediated many-body interactions, and (2) the emergence of an indirect gap in bilayers, which opens a phonon-assisted (momentum-conserving) annihilation pathway that is intrinsically less probable than direct EEA in monolayers.

These mechanisms account for the overall EEA reduction from monolayer to bilayer but do not by themselves explain the additional stacking dependence between 2H and 3R bilayers. First, screened Coulomb interactions in 2D semiconductors are governed by the dielectric environment and the 2D polarizability χ_2D_.^55^ In our experiments, the environment is identical for all regions (same substrate), and χ_2D_ in MoS_2_ scales approximately linearly with layer number, i.e., χ_2D_(bilayer) ≈ 2χ_2D_(monolayer), for both 2H and 3R bilayers.^56,57^ Thus, the enhancement of screening relative to the monolayer should be comparable in 2H and 3R and cannot explain their different γ_EEA_. This is also consistent with microscopic theory indicating that EEA relies strongly on large-momentum Coulomb scattering where environmental screening plays a weaker role than intrinsic TMD polarization.^23^

Second, the phonon-assisted indirect channel should depend primarily on the indirect-gap energy and the relevant phonon phase space. In MoS_2_, 2H and 3R bilayers have similar indirect-gap energies,^34,58^ and their phonon dispersions and density of states are essentially the same over a broad frequency range.^48,59^ The main stacking-dependent phonon differences appear in the low-frequency interlayer shear and breathing modes near the zone center [Figure 2(a)],^48,49^ whereas indirect (intervalley) processes typically require large in-plane momentum transfer and are dominated by finite-momentum intralayer phonons near the zone edge, not zone-center interlayer modes.^60^ Consequently, phonon-mediated contributions to indirect EEA are expected to be broadly comparable in 2H and 3R bilayers under the present room-temperature conditions, although subtle stacking-dependent differences in indirect/phonon-assisted relaxation pathways cannot be fully excluded. This room-temperature comparability is also consistent with prior ultrafast studies in regimes where EEA is not clearly resolved from the measured time window,^35,36^ which report broadly similar 2*H*/3R exciton dynamics dominated primarily by indirect/phonon-assisted relaxation channels. For instance, transient absorption measurements on twisted bilayer MoS_2_ performed at pump fluences ∼30–100 times higher than ours report that the early time decay (on the order of a few ps) is dominated by ultrafast nonradiative channels, often attributed to defect-assisted and/or Auger-like processes, making it difficult to isolate EEA-related kinetics explicitly.^35^ In that high-density regime, the subsequently observed slower dynamics are instead discussed in terms of phonon-assisted exciton relaxation and recombination that depend primarily on interlayer coupling (and thus interlayer distance), which can lead to comparable exciton lifetimes for 2H and 3R stackings.^35^ Moreover, ultrafast spectroscopy measurements on twisted WS_2_ bilayers performed at pump fluences approximately 180 times lower than those used in our study, where EEA is not expected to contribute significantly, have also reported comparable exciton lifetimes for the 2H and 3R configurations.^36^

In contrast, pump–probe measurements performed in a comparatively weak excitation regime (up to ∼30 μJ cm^–2^, i.e., roughly half of our pump fluence) reported that the 3R bilayer exhibits an exciton lifetime approximately twice as long as that of the 2H bilayer, which was attributed to reduced electron–hole wave function overlap in the 3R stacking.^34^ In that low-density regime, the exciton decay shows little fluence dependence, consistent with the expectation that EEA does not contribute significantly, similar to our observations, where the exciton decay traces at 20 and 28 μJ cm^–2^ do not differ markedly (Figure 3). A similar absence of systematic pump-fluence dependence has also been reported even at higher excitation densities (up to pump fluence ∼60 μJ cm^–2^, comparable to our conditions), where fluence-dependent lifetime trends are difficult to resolve.^30^ We attribute this apparent insensitivity, at least in part, to EEA suppression induced by h-BN encapsulation, which can enhance dielectric screening and reduce disorder, thereby hindering the identification of EEA contributions even at relatively high excitation densities.^22,23^

Our intermediate excitation density, combined with the absence of h-BN encapsulation, places the present measurements in a regime where EEA-related exciton dynamics can be resolved within the relevant time window. Although EEA-independent channels, including radiative recombination and phonon-assisted relaxation, also contribute to the transient decay, they are expected to show only weak pump-fluence dependence over our experimental range and to remain broadly similar between the 2H and 3R bilayers.^35,36,58^ The pump-fluence-dependent component of the dynamics therefore primarily reflects EEA, allowing a direct comparison of the EEA rates between the two stackings. The further suppression of EEA observed in the 3R bilayer thus indicates that stacking configuration provides an additional degree of freedom for modulating EEA beyond layer-number effects. A key feature unique to 3R MoS_2_ is its broken inversion symmetry, which generates out-of-plane spontaneous polarization and promotes spatial separation of electrons and holes. The resulting dipolar excitons can significantly influence nonequilibrium exciton dynamics, consistent with previous reports of prolonged exciton lifetimes^34^ and superdiffusive exciton transport^30^ in 3R MoS_2_ bilayers. In our case, such aligned dipole moments can give rise to repulsive dipole–dipole interactions, thereby reducing the probability of the close exciton–exciton encounters required for Auger-type annihilation.^61,62^ Below, we develop a microscopic picture for the reduced EEA in 3R MoS_2_ based on these repulsive dipolar interactions.

### Dipolar Repulsion and EEA Suppression in 3R MoS_2_

The effective bimolecular EEA rate γ_EEA_ can be expressed in terms of a diffusion-limited contribution *k_D_* and a rate-limited contribution *k_R_* as^10^

3 $$\frac{1}{\gamma_{\mathrm{EEA}}}=\frac{1}{k_{D}}+\frac{1}{k_{R}}$$

Here, *k*_*D*_ characterizes mutual diffusion of excitons (*k*_*D*_ ∝ *D*, where *D* is the exciton diffusion coefficient),^11,12^ whereas *k*_*R*_ captures the intrinsic (rate-limited) annihilation rate once two excitons are sufficiently close. For monolayer and multilayer TMDs, overall exciton diffusion is an order of magnitude faster than annihilation,^10^ so that γ_EEA_ is expected to be rate-limited (γ_EEA_ ≈ *k*_*R*_). Our stacking-dependent data further support this view: recent measurements show that *D* in 3R can be an order of magnitude larger than in 2H MoS_2_,^30^ which, under a purely diffusion-limited picture (γ_EEA_ ≈ *k*_*D*_ ∝ *D*), would predict a larger γ_EEA_ in 3R. Instead, we observe the opposite trend (suppressed EEA in 3R relative to 2H), indicating that the intrinsic annihilation probability, rather than diffusive transport, governs the effective EEA rates in our measurements, which is consistent with prior reports of only a weak dependence of γ_EEA_ on exciton diffusion.^10,12^ We note that the previously reported faster exciton diffusion in 3R than 2H bilayers^30^ was obtained from mechanically exfoliated samples, and therefore the absolute diffusion coefficients are not used here as quantitative inputs for the present CVD-grown system. However, we cite the reported diffusivity contrast qualitatively, because the built-in polarization and dipolar exciton character associated with 3R stacking are also intrinsic features of CVD-grown bilayers.^63,64^ We therefore regard the previously reported diffusion trend as qualitatively relevant to the present samples.

Within the rate-limited picture, the repulsive dipolar interactions in 3R MoS_2_ reduce close exciton–exciton encounters as illustrated in Figure 4(a) and thus further suppress EEA compared to 2H MoS_2_, where such dipolar repulsion is absent. For two-body interactions, the exciton–exciton encounter probability distribution, *f*, in the presence of the dipole–dipole potential, *V*_dd_(*r*), can be written as^61,62^

4 $$f=Uf_{0}$$

where *r* is the exciton–exciton separation, *f*_0_ is the probability distribution in the absence of dipolar repulsion, and *U* = e^–*V*_dd_(*r*)/*k*_*B*_ *T*^ is the Boltzmann factor associated with dipolar repulsion, where *k*_B_ is the Boltzmann constant and *T* = 300 K. Here, we take the 2H bilayer as the nonpolar reference, for which the short-range encounter probability is represented by *f*_0_. Assuming the effective EEA rate scales with the close-encounter probability in the rate-limited regime, the EEA rate in 3R relative to 2H is expected to be suppressed by a factor given by

5 $$\frac{\gamma_{\mathrm{EEA},3R}}{\gamma_{\mathrm{EEA},2H}}\approx \frac{f}{f_{0}}=U$$

**Figure 4:**
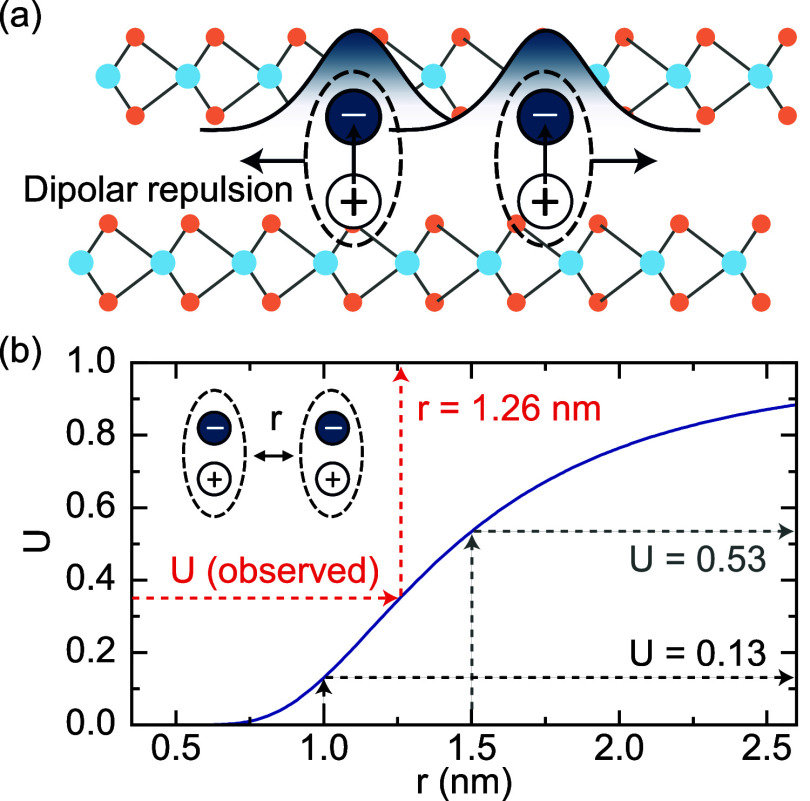
Schematic of EEA suppression in 3R MoS_2_ bilayers. (a) Illustration of dipole–dipole repulsion, where spontaneous polarization promotes layer-polarized, dipolar excitons. (b) Calculated Boltzmann suppression factor, *U* = exp­[−*V*_dd_(*r*)/*k*_*B*_ *T*], as a function of the in-plane exciton–exciton separation *r*. The red dashed line indicates the measured EEA suppression in 3R relative to the nonpolar 2H bilayer and the corresponding separation (*r* ≈ 1.26 nm). The black and gray dashed lines mark plausible separations (*r* = 1.0 and 1.5 nm) and their corresponding suppression factors (*U* = 0.13 and 0.53), respectively.

To model the repulsive dipolar interactions *V*_dd_(*r*), we consider each exciton as an out-of-plane electric dipole arising from the average vertical electron–hole separation (*d*), giving

6 $$V_{\mathrm{dd}}\left(r\right)=\frac{e^{2}}{2\pi \epsilon_{0}\kappa }\left(\frac{1}{r}-\frac{1}{\sqrt{r^{2}+d^{2}}}\right)$$

where κ = (ε_top_ + *ε*_bottom_)/2 is the environmental dielectric constant. For our geometry, ε_top_ = ε_air_ = 1 and *ε*_bottom_ = ε_SiO_2__ = 3.9,^65^ yielding κ = 2.45. In 3R-stacked MoS_2_ bilayers, the electron is localized in one layer while the hole is shared between the two layers [Figure 1(c)]. In other words, the conduction-band minimum at K is layer-selective while the Γ valence states are more strongly interlayer-hybridized. As a result, the average vertical electron–hole separation is approximately half the interlayer spacing (∼0.62 nm),^40^ giving *d* ∼ 0.31 nm and a dipole moment of ∼0.31 *e*·nm.^32^ The resulting Boltzmann suppression factor (*U*) is plotted in Figure 4(b). In the rate-limited framework, annihilation is governed by short-range Auger-like coupling that requires significant wave function overlap,^66,67^ so the relevant interaction length scale is on the order of exciton size. Using a typical exciton Bohr radius of bilayer MoS_2_ (∼1 nm),^68,69^ we obtain *V*_dd_(*r*) = 52.7 meV and *U* = 0.13, with the same order of magnitude as the measured ratio γ_EEA,3R_/γ_EEA,2H_ ≈ 0.35. Notably, the measured ratio is reproduced by *r* ≈ 1.26 nm, which is close to the theoretically predicted effective Bohr radius (∼1.3 nm).^69^ This agreement supports the interpretation of *r* as an effective exciton–exciton interaction length scale. We model the dipolar repulsion using the electrostatic interaction between two out-of-plane dipoles in an effective dielectric environment, which provides an order-of-magnitude estimate for the short-range suppression factor. More refined treatments incorporating 2D screening (e.g., the Rytova–Keldysh potential)^55^ are expected to renormalize *V*_dd_(*r*) but do not alter the central conclusion that dipolar repulsion reduces close-encounter probability and suppresses EEA. Overall, repulsive dipolar interactions generated by spontaneous polarization in 3R MoS_2_ provide a quantitative, physically plausible mechanism for the additional suppression of EEA relative to 2H bilayers, highlighting the role of layer polarization in exciton–exciton annihilation.

## Conclusions

Fluence-dependent ultrafast optical spectroscopy revealed stacking-dependent EEA rates and enabled a direct comparison of annihilation rates in CVD-grown MoS_2_ monolayers and in 2H- and 3R-stacked MoS_2_ bilayers. By analyzing the density-dependent A-exciton bleaching dynamics using a bimolecular recombination model, we extracted EEA rates of (9.14 ± 0.85) × 10^–2^ cm^2^ s^–1^ for the monolayer, (1.43 ± 0.37) × 10^–2^ cm^2^ s^–1^ for the 2H bilayer, and (5.03 ± 0.99) × 10^–3^ cm^2^ s^–1^ for the 3R bilayer. The reduced annihilation rates in the bilayers are attributed to enhanced dielectric screening and the emergence of a phonon-assisted indirect annihilation pathway associated with the indirect band gap of bilayer TMDs. Beyond this layer-number effect, we observed an additional suppression of EEA in 3R MoS_2_ bilayers that is consistent with spontaneous-polarization-induced dipolar interactions associated with inversion-symmetry breaking. The resulting dipolar excitons, formed through out-of-plane electron–hole separation, experience repulsive dipole–dipole interactions that reduce the probability of exciton–exciton encounters. While temperature-dependent spectroscopy and/or theoretical calculations would further support this interpretation, the present room-temperature comparison already establishes a robust stacking-dependent suppression of EEA in 3R bilayers. Prior reports of faster exciton transport in 3R MoS_2_ bilayers^30^ provide qualitative support for the rate-limited interpretation^11,12,70^ adopted here, although those transport results were obtained for different sample platforms and are not used as quantitative inputs for the present CVD-grown bilayers. However, EEA in two-dimensional semiconductors is not universally governed by diffusion alone;^10,12^ it can instead enter a rate-limited regime in which the bottleneck is the short-range Auger-like annihilation probability, determined by microscopic carrier overlap and interaction potentials. Within this framework, enhanced transport does not necessarily imply stronger EEA. Our observation of additional EEA suppression in the polar 3R stacking, together with the quantitative agreement of a dipolar-repulsion model, supports a rate-limited annihilation picture in 3R MoS_2_ bilayers. The EEA rates obtained here should also be useful for modeling optoelectronic device operation, as they provide a quantitative parameter for this additional decay channel.^46^ These findings indicate that stacking configuration and built-in polarization can be leveraged to mitigate exciton–exciton interactions and the associated nonlinear loss processes in two-dimensional semiconductors, thereby supporting higher sustainable exciton densities in 3R bilayers and offering potential advantages for bright emitters, nonlinear optics, and ferroelectric optoelectronic platforms.

## Methods

### Sample Preparation

Bilayer MoS_2_ samples were synthesized by atmospheric-pressure CVD. SiO_2_/Sisubstrates (285 nm oxide thickness) were placed face-down above an alumina crucible containing ∼2 mg of MoO_2_ powder mixed with CsBr in a weight ratio of ∼1:0.04. This mixture was designated as the precursor. The crucible containing the MoO_2_ precursor and substrates was loaded at the center of a quartz tube. Another crucible containing ∼50 mg of sulfur powder was placed upstream, where heating tape was wrapped around the tube. After flushing the tube with 500 standard cubic centimeters per minute (sccm) of ultrahigh-purity argon gas, the precursor was heated to 780 °C (with a ramp rate of 40 °C/min), and the sulfur was heated to ∼ 200 °C by the heating tape under an 80 sccm argon gas flow. The reaction was carried out for 3 min. After growth, the heating tape was removed, and the furnace was opened to allow rapid fan-assisted cooling to room temperature.

### Static Optical Spectroscopy

Raman measurements were conducted using a Jobin-Yvon T64000 triple spectrometer equipped with 1800 grooves/mm gratings and a liquid-nitrogen-cooled CCD detector (Symphony Horiba JY). All measurements were performed at room temperature using a 532 nm continuous-wave laser coupled into an upright microscope in a backscattering configuration. The 532 nm laser beam was focused through a 100 × objective (NA = 0.9), yielding a beam spot of ∼1 μm with an incident laser power of ∼0.3 mW. Linear reflectance spectra (*R*_0_) were measured at the same sample locations using a laser-driven broadband light source (Energetiq Inc.).

### Ultrafast Optical Spectroscopy

Transient reflectance measurements were performed using a Ti:sapphire oscillator (Micra, Coherent) seeding a Ti:sapphire regenerative amplifier (Legend USP-HE, Coherent) operating at a 1 kHz repetition rate. The amplifier delivered ∼57 fs pulses centered at 800 nm with a pulse energy of 1.8 mJ. A broadband white-light continuum probe was generated by focusing a small portion of the 800 nm beam into a 2 mm-thick sapphire window. Pump pulses at 3.1 eV were produced via second-harmonic generation of the 800 nm fundamental. Collinear pump and probe beams were directed into a custom-built upright microscope equipped with a 36 × reflective objective, resulting in pump and probe spot diameters of ∼5 μm at the sample. The pump fluence was adjusted using neutral-density filters. The reflected probe spectrum was dispersed by a spectrograph (Shamrock 303i, Andor) and detected with an EMCCD (Newton, Andor). The pump and probe pulses were cross-polarized, and a polarizer placed before the spectrometer was used to suppress pump scattering in the transient reflectance map. At each pump–probe delay, the transient reflectance was calculated as Δ*R*/*R*_0_ = (*R*_pump_ – *R*_0_)/*R*_0_, where *R*_pump_ and *R*_0_ denote the reflected probe intensities with the pump on and off, respectively.

The initial exciton density per layer, *n*_0_, was estimated using an optical absorption of ∼ 25% per MoS_2_ layer at the pump wavelength.^71^ Substrate interference was accounted for by the field-intensity reduction factor *I*/*I*_0_ = |1 + *r*_eff_|^2^ ≈ 0.1 for 285 nm-thick SiO_2_ on Si at the pump wavelength. Here,*r*_eff_ = (*r*_12_ + *r*_23_Ω_2_)/(1 + *r*_12_Ω_2_) with *r*_*ij*_ = (*ñ*_*i*_ – *ñ*_*j*_)/(*ñ*_*i*_ + *ñ*_*j*_),^72^ where subscripts 1, 2, and 3 denote air, SiO_2_, and Si, respectively. The phase factor is given by Ω_2_ = exp­(−4*πiñ*_2_ *d*_2_/λ_pump_), where *ñ*_2_ and *d*_2_ are the complex refractive index and thickness of the SiO_2_ layer, respectively, and λ_pump_ is the pump wavelength. The complex refractive indices of SiO_2_ and Si were taken from elsewhere.^73,74^

## Supplementary Material



## Notes

Raman spectroscopy of monolayer, 2H-, and 3R-stacked bilayer MoS_2_ (Note S1 and Figure S1); pump–probe signal and exciton–exciton annihilation rates in monolayer regions adjacent to 2H and 3R bilayers (Note S2 and Figure S2); pump-fluence dependence of the pump–probe signal (Note S3 and Figure S3); *F*_pump_-dependent fast and slow decay constants for monolayer, 2H, and 3R bilayer MoS_2_ (Table S1); and exciton–exciton annihilation rates extracted at different *F*_pump_ (Table S2) (PDF)

## References

[1] Mak K. F., Shan J. (2016). Photonics and optoelectronics
of 2D semiconductor transition metal
dichalcogenides. Nat. Photonics.

[2] Wang G., Chernikov A., Glazov M. M., Heinz T. F., Marie X., Amand T., Urbaszek B. (2018). Colloquium: Excitons in atomically
thin transition metal dichalcogenides. Rev.
Mod. Phys..

[3] Cheng Z., Cao R., Wei K., Yao Y., Liu X., Kang J., Dong J., Shi Z., Zhang H., Zhang X. (2021). 2D materials
enabled next-generation integrated optoelectronics: from fabrication
to applications. Adv. Sci..

[4] Autere A., Jussila H., Dai Y., Wang Y., Lipsanen H., Sun Z. (2018). Nonlinear optics with 2D layered materials. Adv. Mater..

[5] de
Abajo F. J. G., Basov D. N., Koppens F. H., Orsini L., Ceccanti M., Castilla S., Cavicchi L., Polini M., Gonçalves P. A. D., Costa A. T. (2025). Roadmap
for photonics with 2D materials. ACS Photon..

[6] Sun D., Rao Y., Reider G. A., Chen G., You Y., Brézin L., Harutyunyan A. R., Heinz T. F. (2014). Observation of rapid exciton-exciton
annihilation in monolayer molybdenum disulfide. Nano Lett..

[7] Kim H., Uddin S. Z., Higashitarumizu N., Rabani E., Javey A. (2021). Inhibited
nonradiative decay at all exciton densities in monolayer semiconductors. Science.

[8] Vosco G., Refaely-Abramson S. (2025). Exciton-Exciton Annihilation Mediated by Many-Body
Coulomb and Phonon Interactions: An Ab Initio Study. Phys. Rev. Lett..

[9] Kumar N., Cui Q., Ceballos F., He D., Wang Y., Zhao H. (2014). Exciton-exciton
annihilation in MoSe_2_ monolayers. Phys. Rev. B.

[10] Yuan L., Huang L. (2015). Exciton dynamics and annihilation in WS_2_ 2D semiconductors. Nanoscale.

[11] Yu Y., Yu Y., Xu C., Barrette A., Gundogdu K., Cao L. (2016). Fundamental
limits of exciton-exciton annihilation for light emission in transition
metal dichalcogenide monolayers. Phys. Rev.
B.

[12] Goodman A. J., Lien D.-H., Ahn G., Spiegel L., Amani M., Willard A., Javey A., Tisdale W. (2020). Substrate-dependent
exciton diffusion and annihilation in chemically treated MoS_2_ and WS_2_. J. Phys. Chem. C.

[13] Chen Y., Cao B., Sun C., Wang Z., Zhou H., Wang L., Zhu H. (2022). Controlling
exciton-exciton annihilation in WSe_2_ bilayers
via interlayer twist. Nano Res..

[14] Li J., Yang R., Higashitarumizu N., Dai S., Wu J., Javey A., Grigoropoulos C. P. (2024). Transient
nanoscopy of exciton dynamics
in 2D transition metal dichalcogenides. Adv.
Mater..

[15] Uddin S. Z., Higashitarumizu N., Kim H., Rahman I. R., Javey A. (2022). Efficiency
roll-off free electroluminescence from monolayer WSe_2_. Nano Lett..

[16] Uddin S. Z., Higashitarumizu N., Kim H., Rabani E., Javey A. (2022). Engineering
exciton recombination pathways in bilayer WSe_2_ for bright
luminescence. ACS Nano.

[17] Arp T. B., Pleskot D., Aji V., Gabor N. M. (2019). Electron-hole liquid
in a van der Waals heterostructure photocell at room temperature. Nat. Photonics.

[18] Yu Y., Bataller A. W., Younts R., Yu Y., Li G., Puretzky A. A., Geohegan D. B., Gundogdu K., Cao L. (2019). Room-temperature
electron-hole liquid in monolayer MoS_2_. ACS Nano.

[19] Yu Y., Li G., Xu Y., Hu C., Liu X., Cao L. (2023). Phase Diagram
of High-Temperature Electron-Hole Quantum Droplet in Two-Dimensional
Semiconductors. ACS Nano.

[20] Sortino L., Gülmüs M., Tilmann B., de S Menezes L., Maier S. A. (2023). Radiative suppression
of exciton-exciton annihilation
in a two-dimensional semiconductor. Light Sci.
Appl..

[21] Xu Y., Xiang Y., Shi M., Zhai B., Dai W., Wang T., Liu X., Yu Y., He J. (2025). Room-Temperature
Amplified Spontaneous Emission in Two-Dimensional WS_2_ beyond
Exciton Mott Transition. Phys. Rev. Lett..

[22] Hoshi Y., Kuroda T., Okada M., Moriya R., Masubuchi S., Watanabe K., Taniguchi T., Kitaura R., Machida T. (2017). Suppression
of exciton-exciton annihilation in tungsten disulfide monolayers encapsulated
by hexagonal boron nitrides. Phys. Rev. B.

[23] Steinhoff A., Jahnke F., Florian M. (2021). Microscopic
theory of exciton-exciton
annihilation in two-dimensional semiconductors. Phys. Rev. B.

[24] Yuan L., Jeong J., Chi Kwock K. W., Yanev E. S., Grandel M., Rhodes D. A., Luk T. S., Schuck P. J., Yarotski D., Hone J. C. (2021). Manipulation
of exciton dynamics in single-layer WSe_2_ using a toroidal
dielectric metasurface. Nano Lett..

[25] Cai C.-S., Lai W.-Y., Liu P.-H., Chou T.-C., Liu R.-Y., Lin C.-M., Gwo S., Hsu W.-T. (2024). Ultralow Auger-Assisted
Interlayer Exciton Annihilation in WS_2_/WSe_2_ Moiré
Heterobilayers. Nano Lett..

[26] Unuchek D., Ciarrocchi A., Avsar A., Sun Z., Watanabe K., Taniguchi T., Kis A. (2019). Valley-polarized exciton currents
in a van der Waals heterostructure. Nat. Nanotechnol..

[27] Li W., Lu X., Dubey S., Devenica L., Srivastava A. (2020). Dipolar interactions
between localized interlayer excitons in van der Waals heterostructures. Nat. Mater..

[28] Sun Z., Ciarrocchi A., Tagarelli F., Marin J. F. G., Watanabe K., Taniguchi T., Kis A. (2022). Excitonic transport driven by repulsive
dipolar interaction in a van der Waals heterostructure. Nat. Photonics.

[29] Yuan L., Zheng B., Zhao Q., Kempt R., Brumme T., Kuc A. B., Ma C., Deng S., Pan A., Huang L. (2023). Strong dipolar repulsion of one-dimensional interfacial
excitons
in monolayer lateral heterojunctions. ACS Nano.

[30] Agunbiade G. S., Rafizadeh N., Zheng T., Zhao H. (2025). Fast Superdiffusive
Transport of Dipolar Excitons in 3R-Stacked MoS_2_ Bilayers. ACS Nano.

[31] Yang D., Wu J., Zhou B. T., Liang J., Ideue T., Siu T., Awan K. M., Watanabe K., Taniguchi T., Iwasa Y. (2022). Spontaneous-polarization-induced
photovoltaic effect
in rhombohedrally stacked MoS_2_. Nat.
Photonics.

[32] Liang J., Yang D., Wu J., Dadap J. I., Watanabe K., Taniguchi T., Ye Z. (2022). Optically probing the asymmetric
interlayer coupling in rhombohedral-stacked MoS_2_ bilayer. Phys. Rev. X.

[33] Dong Y., Yang M.-M., Yoshii M., Matsuoka S., Kitamura S., Hasegawa T., Ogawa N., Morimoto T., Ideue T., Iwasa Y. (2023). Giant bulk piezophotovoltaic effect
in 3R-MoS_2_. Nat. Nanotechnol..

[34] Agunbiade G., Rafizadeh N., Scott R. J., Zhao H. (2024). Transient absorption
measurements of excitonic dynamics in 3R-MoS_2_. Phys. Rev. B.

[35] Zhou J., Zhang H., Li L., Peng Z., Yao B., Yu Y., Li Z., Xu Q., Luo W., Deng C. (2026). Oxygen-Assisted Direct Synthesis of Twisted Bilayer
MoS_2_ with Tunable Exciton Lifetime. ACS Nano.

[36] Zhong Y., Yue S., Liang J., Yuan L., Xia Y., Tian Y., Zheng Y., Zhang Y., Du W., Li D. (2025). Twist
angle-dependent exciton mobility in WS_2_ bilayers. Nano Lett..

[37] Liu H., Wang C., Liu D., Luo J. (2019). Neutral and defect-induced
exciton annihilation in defective monolayer WS_2_. Nanoscale.

[38] Soni A., Kamath N. S., Shen Y.-Y., Seksaria H., De Sarkar A., Chang W.-H., Pal S. K. (2025). Substrate-induced modulation of transient
optical response of large-area monolayer MoS_2_. Sci. Rep..

[39] Zhang T., Wang J. (2021). Defect-enhanced exciton-exciton
annihilation in monolayer transition
metal dichalcogenides at high exciton densities. ACS Photon..

[40] He J., Hummer K., Franchini C. (2014). Stacking effects on the electronic
and optical properties of bilayer transition metal dichalcogenides
MoS_2_, MoSe_2_, WS_2_, and WSe_2_. Phys. Rev. B.

[41] Paradisanos I., Shree S., George A., Leisgang N., Robert C., Watanabe K., Taniguchi T., Warburton R. J., Turchanin A., Marie X. (2020). Controlling
interlayer
excitons in MoS_2_ layers grown by chemical vapor deposition. Nat. Commun..

[42] Shi J., Yu P., Liu F., He P., Wang R., Qin L., Zhou J., Li X., Zhou J., Sui X. (2017). 3R MoS_2_ with
broken inversion symmetry: a promising ultrathin
nonlinear optical device. Adv. Mater..

[43] Zhang D., Zeng Z., Tong Q., Jiang Y., Chen S., Zheng B., Qu J., Li F., Zheng W., Jiang F. (2020). Near-unity polarization
of valley-dependent second-harmonic
generation in stacked TMDC layers and heterostructures at room temperature. Adv. Mater..

[44] Li X., Qin B., Wang Y., Xi Y., Huang Z., Zhao M., Peng Y., Chen Z., Pan Z., Zhu J. (2024). Sliding ferroelectric memories and synapses
based on rhombohedral-stacked
bilayer MoS_2_. Nat. Commun..

[45] Yang D., Liang J., Wu J., Xiao Y., Dadap J. I., Watanabe K., Taniguchi T., Ye Z. (2024). Non-volatile electrical
polarization switching via domain wall release in 3R-MoS_2_ bilayer. Nat. Commun..

[46] Wu J., Yang D., Liang J., Werner M., Ostroumov E., Xiao Y., Watanabe K., Taniguchi T., Dadap J. I., Jones D., Ye Z. (2022). Ultrafast response
of spontaneous photovoltaic effect in 3R-MoS_2_-based heterostructures. Sci. Adv..

[47] Liang J., Xie Y., Yang D., Guo S., Watanabe K., Taniguchi T., Dadap J. I., Jones D., Ye Z. (2025). Nanosecond Ferroelectric
Switching of Intralayer Excitons in Bilayer 3R-MoS_2_ through
Coulomb Engineering. Phys. Rev. X.

[48] Huang S., Liang L., Ling X., Puretzky A. A., Geohegan D. B., Sumpter B. G., Kong J., Meunier V., Dresselhaus M. S. (2016). Low-frequency
interlayer Raman modes to probe interface of twisted bilayer MoS_2_. Nano Lett..

[49] Yan J., Xia J., Wang X., Liu L., Kuo J.-L., Tay B. K., Chen S., Zhou W., Liu Z., Shen Z. X. (2015). Stacking-dependent
interlayer coupling in trilayer MoS_2_ with broken inversion
symmetry. Nano Lett..

[50] Niu Y., Gonzalez-Abad S., Frisenda R., Marauhn P., Drüppel M., Gant P., Schmidt R., Taghavi N. S., Barcons D., Molina-Mendoza A. J. (2018). Thickness-dependent differential reflectance
spectra of monolayer and few-layer MoS_2_, MoSe_2_, WS_2_ and WSe_2_. Nanomaterials.

[51] Nie Z., Long R., Sun L., Huang C.-C., Zhang J., Xiong Q., Hewak D. W., Shen Z., Prezhdo O. V., Loh Z.-H. (2014). Ultrafast carrier thermalization and cooling dynamics
in few-layer MoS_2_. ACS Nano.

[52] Wang H., Zhang C., Rana F. (2015). Ultrafast
dynamics of defect-assisted
electron-hole recombination in monolayer MoS_2_. Nano Lett..

[53] Das S., Wang Y., Dai Y., Li S., Sun Z. (2021). Ultrafast
transient sub-bandgap absorption of monolayer MoS_2_. Light Sci. Appl..

[54] Tsai H.-S., Huang Y.-H., Tsai P.-C., Chen Y.-J., Ahn H., Lin S.-Y., Lu Y.-J. (2020). Ultrafast
exciton dynamics in scalable
monolayer MoS_2_ synthesized by metal sulfurization. ACS Omega.

[55] Cudazzo P., Tokatly I. V., Rubio A. (2011). Dielectric screening
in two-dimensional
insulators: Implications for excitonic and impurity states in graphane. Phys. Rev. B.

[56] Tian T., Scullion D., Hughes D., Li L. H., Shih C.-J., Coleman J., Chhowalla M., Santos E. J. (2020). Electronic polarizability
as the fundamental variable in the dielectric properties of two-dimensional
materials. Nano Lett..

[57] Ferreira F., Enaldiev V., Fal’ko V. (2022). Scaleability
of dielectric susceptibility *ϵ*
_
*zz*
_ with the number of
layers and additivity of ferroelectric polarization in van der Waals
semiconductors. Phys. Rev. B.

[58] Liu K., Zhang L., Cao T., Jin C., Qiu D., Zhou Q., Zettl A., Yang P., Louie S. G., Wang F. (2014). Evolution of interlayer coupling
in twisted molybdenum disulfide
bilayers. Nat. Commun..

[59] Coutinho S., Tavares M., Barboza C., Frazão N., Moreira E., Azevedo D. L. (2017). 3R and 2H polytypes
of MoS_2_: DFT and DFPT calculations of structural, optoelectronic,
vibrational
and thermodynamic properties. J. Phys. Chem.
Solids.

[60] Hu J., Xiang Y., Ferrari B. M., Scalise E., Vanacore G. M. (2023). Indirect
exciton-phonon dynamics in MoS_2_ revealed by ultrafast electron
diffraction. Adv. Funct. Mater..

[61] Knorr N., Brune H., Epple M., Hirstein A., Schneider M., Kern K. (2002). Long-range adsorbate interactions mediated by a two-dimensional electron
gas. Phys. Rev. B.

[62] Yokoyama T., Takahashi T., Shinozaki K., Okamoto M. (2007). Quantitative Analysis
of Long-Range Interactions between Adsorbed Dipolar Molecules on Cu
(111). Phys. Rev. Lett..

[63] Jiang H., Li L., Wu Y., Duan R., Yi K., Wu L., Zhu C., Luo L., Xu M., Zheng L. (2024). Vapor
deposition of bilayer 3R MoS_2_ with room-temperature ferroelectricity. Adv. Mater..

[64] Fan A., Zhang Q., Yang Z., Li L., Li M., Zhang K., Gao J., Wu F., Wu M., Geng D., Hu W. (2025). Tailored sliding ferroelectricity
for ultrahigh fatigue resistance in stacked trilayer MoS_2_ crystals. Sci. Adv..

[65] Robertson J. (2004). High dielectric
constant oxides. Eur. Phys. J. Appl. Phys..

[66] Erkensten D., Brem S., Wagner K., Gillen R., Perea-Causín R., Ziegler J. D., Taniguchi T., Watanabe K., Maultzsch J., Chernikov A., Malic E. (2021). Dark exciton-exciton annihilation
in monolayer WSe_2_. Phys. Rev. B.

[67] Erkensten D., Brem S., Malic E. (2021). Exciton-exciton
interaction in transition
metal dichalcogenide monolayers and van der Waals heterostructures. Phys. Rev. B.

[68] Yu Y., Yu Y., Cai Y., Li W., Gurarslan A., Peelaers H., Aspnes D. E., Van de Walle C. G., Nguyen N. V., Zhang Y.-W., Cao L. (2015). Exciton-dominated dielectric
function of atomically thin MoS_2_ films. Sci. Rep..

[69] Cheiwchanchamnangij T., Lambrecht W. R. (2012). Quasiparticle
band structure calculation of monolayer,
bilayer, and bulk MoS_2_. Phys. Rev.
B.

[70] Mouri S., Miyauchi Y., Toh M., Zhao W., Eda G., Matsuda K. (2014). Nonlinear photoluminescence
in atomically thin layered
WSe_2_ arising from diffusion-assisted exciton-exciton annihilation. Phys. Rev. B.

[71] Canales A., Kotov O., Shegai T. O. (2023). Perfect absorption
and strong coupling
in supported MoS_2_ multilayers. ACS
Nano.

[72] Li S.-L., Miyazaki H., Song H., Kuramochi H., Nakaharai S., Tsukagoshi K. (2012). Quantitative Raman spectrum and reliable
thickness identification for atomic layers on insulating substrates. ACS Nano.

[73] Rodríguez-de
Marcos L. V., Larruquert J. I., Méndez J. A., Aznárez J. A. (2016). Self-consistent optical constants of SiO_2_ and Ta_2_O_5_ films. Opt.
Mater. Express.

[74] Aspnes D. E., Studna A. (1983). Dielectric functions and optical parameters of Si,
Ge, GaP, GaAs, GaSb, InP, InAs, and InSb from 1.5 to 6.0 eV. Phys. Rev. B.

