# Supplementary material for: Spontaneous Polarization Suppression of Exciton–Exciton Annihilation in Rhombohedral-Stacked Bilayer Molybdenum Disulfide
Source: ACS Nano. 2026 Jun 25;20(26):18873–82. doi: 10.1021/acsnano.6c05069 (PMC13348177; doi:10.1021/acsnano.6c05069)
Supplement: Supplementary file 1 [file nn6c05069_si_001.pdf]

Supporting Information for

Spontaneous Polarization Suppression of Exciton-Exciton Annihilation in Rhombohedral-Stacked Bilayer Molybdenum Disulfide

Tae Gwan Park,[†,‡] Xufan Li,[¶] Kyungnam Kang,[†] David B. Geohegan,[§] Christopher M. Rouleau,[†] Alexander A. Puretzky,[*,†] and Kai Xiao[*,†]

†*Center for Nanophase Materials Sciences, Oak Ridge National Laboratory, Oak Ridge, Tennessee 37831, USA*

‡*Center for Integrated Nanotechnologies, Los Alamos National Laboratory, Los Alamos, New Mexico 87544, USA*

¶*Honda Research Institute USA Inc., San Jose, California 95134, USA*

§*Department of Materials Science and Engineering, University of Tennessee at Knoxville, Knoxville, Tennessee 37996, USA*

E-mail: puretzkya@ornl.gov; xiaok@ornl.gov

Note S1: Raman spectroscopy of monolayer and bilayer MoS_2 with 2H and 3R stacking

Figure S1 presents Raman spectra of MoS_2 monolayers and bilayers with different stacking configurations. As discussed in the main text, the low-frequency modes below 60 cm^{-1} originate from interlayer vibrations, namely the shear and breathing modes (SM and BM). These modes therefore provide a direct fingerprint of bilayer formation and stacking-dependent interlayer coupling. In the higher-frequency region, the characteristic intralayer phonons E^1_{2g} and A_{1g} are observed for all samples. Notably, the monolayer spectra measured alongside the 2H and 3R bilayers are nearly identical in both peak positions and line shapes, indicating comparable crystal quality for the 2H and 3R samples. In bilayers, we observe the expected softening of the E^1_{2g} mode and stiffening of the A_{1g} mode compared to the monolayer as the number of layers increases, consistent with prior reports.[1]

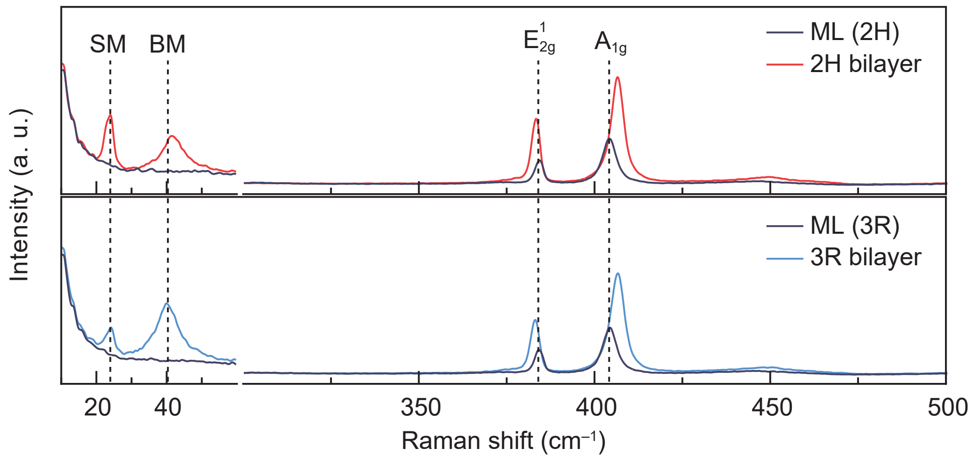

Figure S1. Raman spectra of monolayer and bilayer MoS_2 with different stacking configurations. *Top:* Raman spectra of a monolayer and a 2H-stacked bilayer. *Bottom:* Raman spectra of a monolayer and a 3R-stacked bilayer. The low-frequency region (< 60 cm^{-1}) highlights the interlayer vibrational modes, including the in-plane shear mode (SM) and out-of-plane breathing mode (BM). The high-frequency region shows the first-order intralayer modes E^1_{2g} and A_{1g}. In each panel, the monolayer spectrum was acquired from the region corresponding to the adjacent bilayer sample.

Note S2: Pump-probe signal and exciton-exciton annihilation rates in monolayer regions adjacent to 2H and 3R bilayers

Figure S2 compares the intrinsic ultrafast recombination kinetics of monolayer MoS_2 measured in the vicinity of the 2H- and 3R-stacked bilayer domains investigated in this work. The $\Delta R/R_0$ traces measured from the monolayer regions adjacent to each bilayer are nearly identical over the pump-probe delay window, indicating comparable carrier/exciton relaxation pathways in the two reference areas. Consistently, the exciton-exciton annihilation (EEA) analysis yields nearly the same slopes in plots of $n_0/n(t) - 1$ versus time, implying that the extracted EEA rates are essentially identical. Because the EEA rate in monolayer MoS_2 is sensitive to local strain and defects,[2–6] and thus to overall crystal quality, this agreement indicates that the 2H and 3R samples possess comparable crystalline quality. Consequently, differences in EEA rates measured for the 2H and 3R bilayers can be attributed to stacking-dependent effects rather than artifacts arising from variations in sample quality.

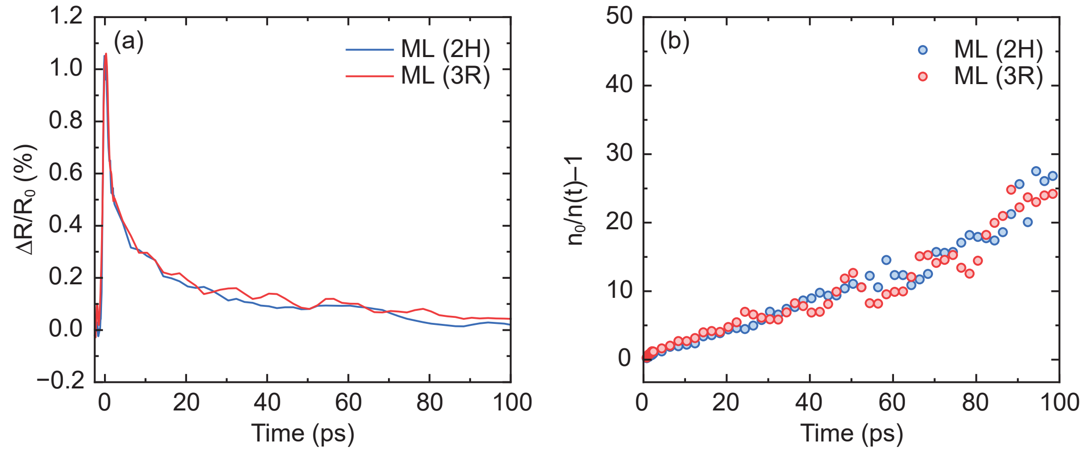

Figure S2. Pump-probe signal and exciton-exciton annihilation rates in monolayer regions adjacent to 2H- and 3R-stacked bilayers. (a) Transient reflectance kinetics measured on monolayer regions adjacent to the 2H and 3R bilayers. (b) Corresponding exciton population analysis plotted as $n_0/n(t) - 1$ versus time. The slope indicates the exciton-exciton annihilation (EEA) rate. The nearly identical transients and comparable slopes indicate similar EEA kinetics in the two monolayer reference regions.

Note S3: Pump-fluence dependence of the pump-probe signal

Figure S3 summarizes the excitation-density dependence of the pump-probe response for all samples studied. For monolayer, 2H bilayer, and 3R bilayer, the peak $\Delta R/R_0$ of the exciton-bleaching signal increases approximately linearly with pump fluence over the measured range, as confirmed by linear fits. This near-linear scaling indicates that the measurements were performed in a regime where the transient-reflectance amplitude is proportional to the photoexcited population, and that no pronounced saturation occurs under our experimental conditions.

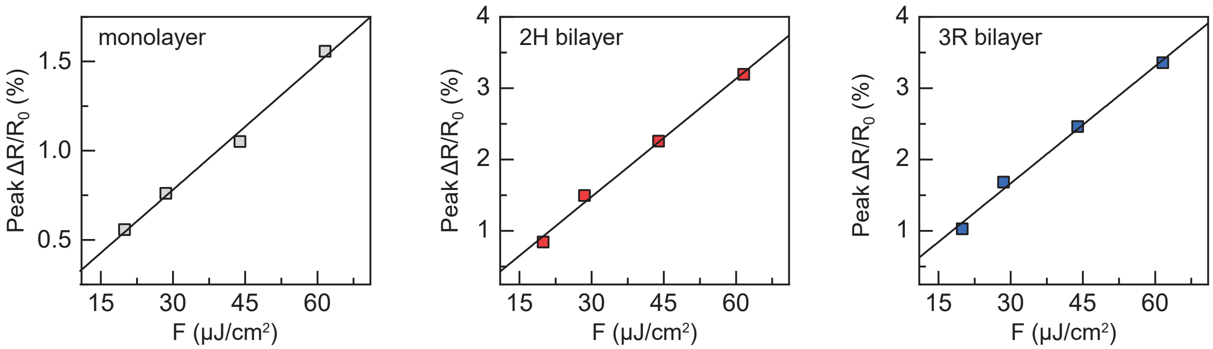

Figure S3. Pump-fluence dependence of the peak pump-probe signal. Peak $\Delta R/R_0$ as a function of pump fluence for monolayer and 2H- and 3R-stacked bilayers. Solid lines are linear fits.

Table 1. F_{pump}-dependent fast and slow decay constants for monolayer and 2H- and 3R-stacked bilayer MoS_2.

	τ_1 (ps)			τ_2 (ps)		
F_{pump} (μJ/cm^2)	ML	2H	3R	ML	2H	3R
61.6	1.0	1.6	1.7	19	64	135
43.9	1.0	1.9	1.8	23	71	147
28.5	0.7	1.2	1.9	27	83	153
20.0	0.8	0.8	0.7	37	95	160

Table 2. Exciton-exciton annihilation (EEA) rates (γ_{EEA}) extracted at different F_{pump} for the monolayer and the 2H and 3R bilayers. For each F_{pump}, γ_{EEA} was obtained from a linear fit of the bleach kinetics described in the main text. The typical fitting uncertainty for an individual γ_{EEA} is $\sim$2%. The average rate corresponds to the mean γ_{EEA} over the investigated fluence range, and the uncertainty is the standard deviation across these values.

F_{pump} (μJ/cm^2)	ML	2H	3R
	γ_{EEA} (cm^2/s)		
61.6	0.0815	0.0140	0.0038
43.9	0.0922	0.0141	0.0047
28.5	0.0932	0.0182	0.0055
20.0	0.0914	0.0107	0.0061
average γ_{EEA}	0.0914 $\pm$ 0.0085	0.0143 $\pm$ 0.0037	0.00503 $\pm$ 0.0099

References

(1) Li, H.; Zhang, Q.; Yap, C. C. R.; Tay, B. K.; Edwin, T. H. T.; Olivier, A.; Baillargeat, D. From bulk to monolayer MoS_2: evolution of Raman scattering. *Adv. Funct. Mater.* **2012**, *22*, 1385–1390.

(2) Kim, H.; Uddin, S. Z.; Higashitarumizu, N.; Rabani, E.; Javey, A. Inhibited nonradiative decay at all exciton densities in monolayer semiconductors. *Science* **2021**, *373*, 448–452.

(3) Uddin, S. Z.; Higashitarumizu, N.; Kim, H.; Rabani, E.; Javey, A. Engineering exciton recombination pathways in bilayer WSe_2 for bright luminescence. *ACS Nano* **2022**, *16*, 1339–1345.

(4) Uddin, S. Z.; Higashitarumizu, N.; Kim, H.; Rahman, I. R.; Javey, A. Efficiency roll-off free electroluminescence from monolayer WSe_2. *Nano Lett.* **2022**, *22*, 5316–5321.

(5) Liu, H.; Wang, C.; Liu, D.; Luo, J. Neutral and defect-induced exciton annihilation in defective monolayer WS_2. *Nanoscale* **2019**, *11*, 7913–7920.

(6) Soni, A.; Kamath, N. S.; Shen, Y.-Y.; Seksaria, H.; De Sarkar, A.; Chang, W.-H.; Pal, S. K. Substrate-induced modulation of transient optical response of large-area monolayer MoS_2. *Sci. Rep.* **2025**, *15*, 7537.
